# MicroRNA-101 inhibits proliferation, migration and invasion of human glioblastoma by targeting SOX9

**DOI:** 10.18632/oncotarget.13706

**Published:** 2016-11-30

**Authors:** Nan Liu, Lei Zhang, Zhen Wang, Yingduan Cheng, Pengxing Zhang, Xin Wang, Weihong Wen, Hongwei Yang, Hui Liu, Weilin Jin, Yongsheng Zhang, Yanyang Tu

**Affiliations:** ^1^ Department of Experimental Surgery, Tangdu Hospital, Fourth Military Medical University, Xi’an 710038, China; ^2^ Department of Research Office, Cipher Ground, North Brunswick, NJ 08902, USA; ^3^ Department of Neurosurgery, Brigham and Women's Hospital, Harvard Medical School, Boston, MA 02115, USA; ^4^ Department of Immunology, Fourth Military Medical University, Xi’an 710032, China; ^5^ Institute of Nano Biomedicine and Engineering, Department of Instrument Science and Engineering, Key Laboratory for Thin Film and Microfabrication Technology of Ministry of Education, School of Electronic Information and Electronic Engineering, Shanghai Jiao Tong University, Shanghai 200240, China; ^6^ Department of Orthopedics, Xi’an Children's Hospital, Xi’an 710003, China

**Keywords:** MiR-101, SOX9, invasion, migration, proliferation

## Abstract

Glioblastoma multiforme (GBM) is the most common primary malignant tumors originating in the brain parenchyma. At present, GBM patients have a poor prognosis despite the continuous progress in therapeutic technologies including surgery, radiotherapy, photodynamic therapy, and chemotherapy. Recent studies revealed that miR-101 was remarkably down-regulated in kinds of human cancers and was associated with aggressive tumor cell proliferation and stem cell self-renewal. Data also showed that miR-101 was down-regulated in primary glioma samples and cell lines, but the underlying molecular mechanism of the deregulation of miR-101 in glioma remained largely unknown. In this study, we found that miR-101 could inhibit the proliferation and invasion of glioma cells both *in vitro* and *in vivo* by directly targeting SOX9 [sex-determining region Y (SRY)-box9 protein]. Silencing of SOX9 exerted similar effects with miR-101 overexpression on glioma cells proliferation and invasion. Quantitative reverse transcription PCR and Western blotting analysis revealed a negative relationship between miR-101 and SOX9 in human glioma U251MG and U87MG cells, and the luciferase assay indicated that miR-101 altered SOX9 expression by directly targeting on 3′UTR. Taken together, our findings suggest that miR-101 regulates glioma proliferation, migration and invasion via directly down-regulating SOX9 both *in vitro* and *in vivo*, and miR-101 may be a potential therapeutic target for future glioma treatment.

## INTRODUCTION

Glioma is the most frequent and malignant brain tumor, which usually originates from neural mesenchymal cells and can be classified into astrocytoma, glioblastoma, medulloblastoma, ependymoma, and oligodendroglioma [1]. Glioma is also divided into four grades according to the WHO classification system, each grade includes varieties of pathological subtypes [2]. Nowadays, the prognosis of traditional treatments such as surgery and radiation therapy is poor [3–7], resulting in low cure rate and short lifetime. The 5-year survival rate of glioblastoma is less than 5%, and the average survival period is only 14 months [8].

Glioblastoma is the highest grade of glioma. It shows low sensitive to radiation therapy and chemotherapy, with poor prognosis and high recurrence rate [9–11]. At present, the international standard treatment of Glioblastoma is temozolomide (TMZ) synchronous chemotherapy after surgical resection, following by cycle specific chemotherapy by TMZ [12, 13]. However, because of the strong radiotherapy and chemotherapy resistance, only 30% patients’ median survival period could reach 2 years, among whom only 9.8% could reach 5 years [14]. Theoretically the microRNA (miRNA) exhibits biological function of glioma occurrence inhibition at the molecular level and improve the survival rate. Studies have also shown that the knockout of miR-21 could inhibit glioblastoma (GBM) proliferation and induce glioma cells apoptosis [15, 16]. These findings provide a potential new strategy for clinical treatment of glioma.

MiRNA is a small endogenous single-stranded RNA (19-25nt) [17] without open reading frame. As a non-coding nucleotide, it plays a vital role in the process of many important life activities and diseases [18]. MiRNA regulates the expression of target genes in transcription level through partial-complementary with its 3′-untranslated region [19]. MiRNA participates in individual development, cell proliferation, differentiation and apoptosis, and other important life activities. All those activities mentioned above have great significance in the occurrence and development of glioma [20]. For example, miR-21, miR-7, miR-128 and miR-221/22 are involved in the progression of gliomas [21, 22]. Several researches have shown that miR-101 was remarkly downregulated in samples from patients and cell lines of human cancers such as lung cancer [28], breast cancer [29], laryngeal squamous cell carcinoma [30], embryonal rhabdomyosarcoma [31] and glioblastoma [32]. In this study, we performed functionally investigation of miR-101 in GBM. Our data suggested that miR-101 regulated glioma cells proliferation and invasion both *in vitro* and *in vivo* by directly targeted SOX9. Simultaneously, SOX9 was proved to be essential for glioma progression. These findings make miR-101 as a new target for glioma therapy and verify the importance of SOX9 in glioma tumorigenesis.

## RESULTS

### Overexpression of miR-101 inhibits glioma cell invasion, migration, and proliferation *in vitro*

**Figure 1:**
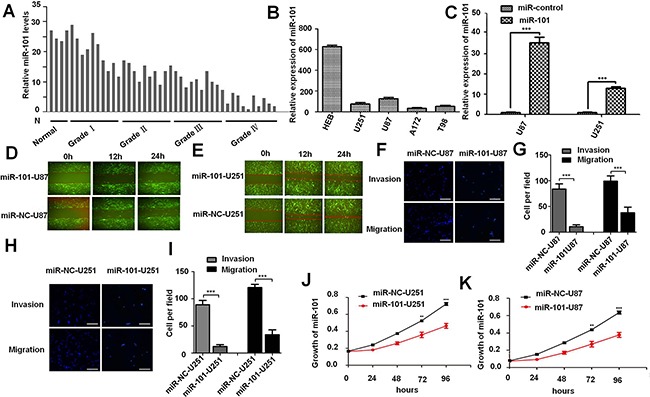
Overexpression of miR-101 inhibits glioma cell invasion, migration, and proliferation *in vitro* **A**. miR-101 expression levels in normal brain tissue and glioma tumor tissues were detected by qRT-PCR analysis. **B**. The expression level of miR-101 in U87MG, U251MG, A172 and T98 glioma cells were detected by qRT-PCR analysis. **C**. The efficiency of lentivirus in U87MG and U251MG glioma cell lines were detected by qRT-PCR analysis. **D. and E**. Representative images of wound healing assay detecting cell migration in miR-101-U87MG, NC-U87MG, miR-101-U251MG and NC-U251MG cells. **F**. Cell invasion and migration of miR-101-U87MG and NC-U87MG cells were detected by trans-well assay. G. Quantitative analysis of the cell number of U87MG cell trans-well invasion and migration assay. **H**. Cell invasion and migration of miR-101-U251MG and NC-U251MG cells were detected by trans-well assay. **I**. Quantitative analysis of the cell number of U251MG cell trans-well invasion and migration assay. **J. and K**. Growth curves of miR-101-U251MG and NC-U251MG, miR-101-U87MG and NC-U87MG cell lines.

In order to test the expression level of miR-101 in glioma, we collected 20 clinical specimens, including 10 grade IV cases, 10 grade III cases, 10 grade II cases, 10 grade I cases and 4 cases of brain injury brain tissues. Our data suggested that miR-101 level in glioma tissue is much lower compared to normal brain tissue (Figure 1A),and the expression level of miR-101 in U87MG, U251MG, A172 and T98 glioma cells are lower than that of HEB cells (Figure 1B). To test miR-101 function in glioma cell lines, U87MG and U251MG were infected with a lentivirus encoding the mature sequence of miR-101. The infected cells expressed high level of miR-101 both in U87MG and U251MG glioma cells (Figure 1C). Wound-healing assay was performed to detect the effect of miR-101 on cell migration, and the miR-101-overexpressing cells showed considerably slower migration compared with the miR-control cells either in U87MG and U251MG glioma cells lines (Figure 1D and 1E). Furthermore, Tran-swell migration assays illustrated that overexpression of miR-101 significantly suppressed glioma cell migration and invasion (Figure 1F-1I). The invasion ability of miR-101-U87MG cells was only approximately one-eighth of miR-control-U87MG cells, and the migration ability was reduced to two-fifth by miR-101-U87MG compared with that of miR-control cells (Figure 1F and 1G). The invasion ability of miR-101-U251MG cell was only approximately one-ninth of miR-control-U251MG cells, and the migration ability was reduced to two-fifth by miR-101-U251MG compared with that of miR-control cells (Figure 1H and 1I). MTT assays were taken to examine the effect of miR-101 on cell proliferation. As shown in Figure 1J and 1K, the cell proliferation rate was decreased in the miR-101 group compared with that of the control group at 24 to 96 hours after transfection (*P < 0.05 for each) in both U87MG and U251MG glioma cells lines, indicating that miR-101 could significantly inhibit the glioma proliferation.

### Overexpression of miR-101 suppresses the tumor growth *in vivo*

**Figure 2:**
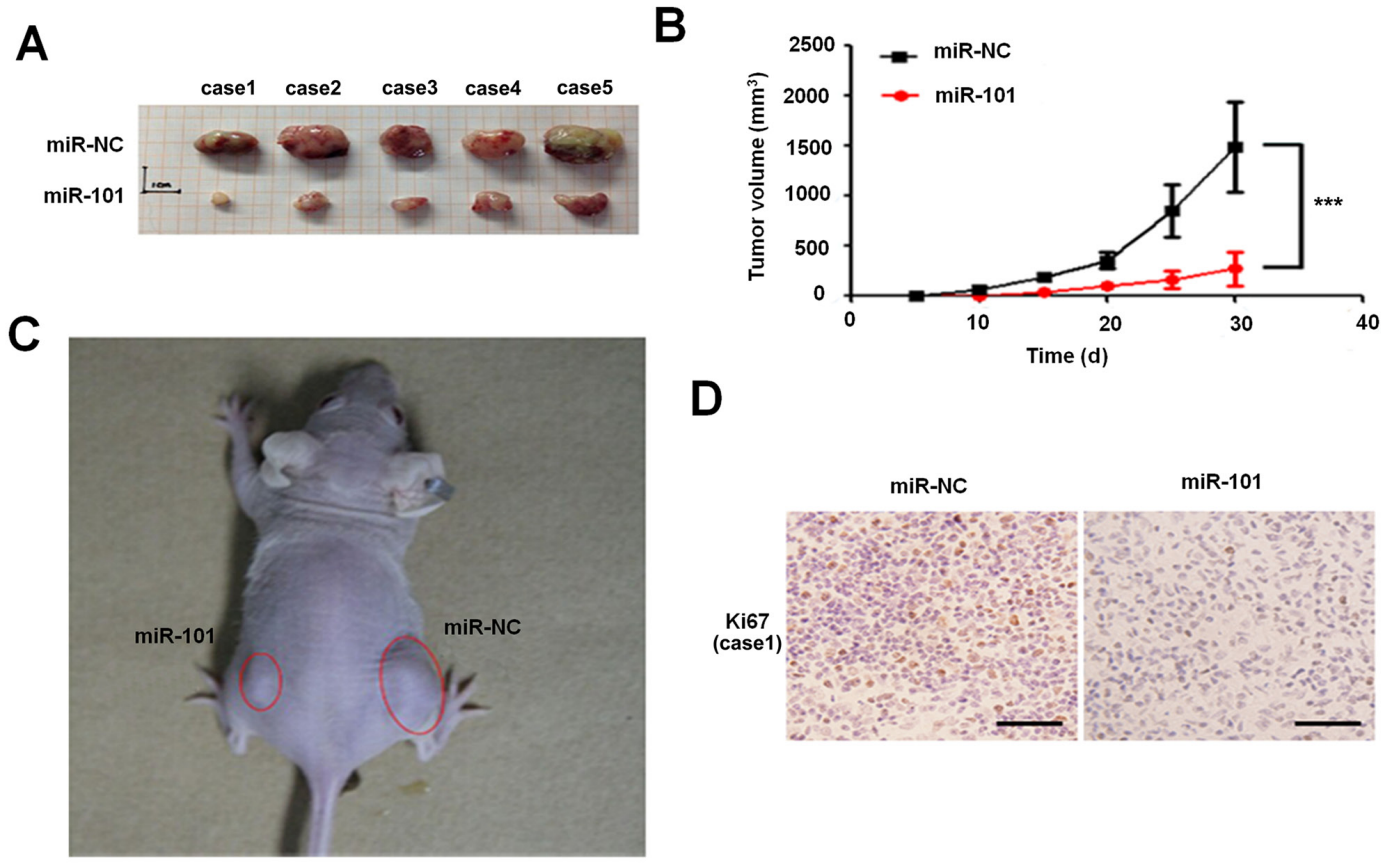
Overexpression of miR-101 suppresses the tumor growth *in vivo* **A. and B**. Determination of the tumor growth, tumor volume was calculated every five days after injection (n = 5). ^**^*p < 0.001. **C**. Representative image for tumor growth is shown. Nude mice were subcutaneously injected with 3.0×10^6^ cells per flank miR-101 or miR-NC stable transfected U87MG cells. **D**. Immunohistochemistry assay detected the level of Ki67 in overexpression-miR-101 and miR-NC xenograft tumor tissues, 200 ×. Scale bar = 100 μm.

In order to investigate the role of miR-101 in glioma, we further tested the effect of miR-101 overexpression on tumor growth *in vivo*. According to the previous study, we chose U87MG which was easy to form xenograft tumors [24]. The miR-101-U87MG cells and their respective control cells were implanted into the left and right flanks (3.0×10^6^ cells per flank) of nude mice by subcutaneous injection, respectively. At 30 days post-injection, data showed that the mean volumes of xenograft tumors of miR-101-U87 cells were significantly smaller than that of miR-control-U87MG cells (*n* = 5 animals per group, *P* = 2.89×10^−3^; Figure 2A, 2B and 2C). Immunohistochemical staining results showed that the number of Ki67 positive cells in miR-101-U87MG tumors was less than that in miR-control-U87MG tumors (Figure 2D and Supplementary Figure 1). Thus, miR-101 overexpression significantly inhibited the glioma proliferation both *in vitro* and *in vivo*.

### MiR-101 directly targets SOX9 in GBM

**Figure 3:**
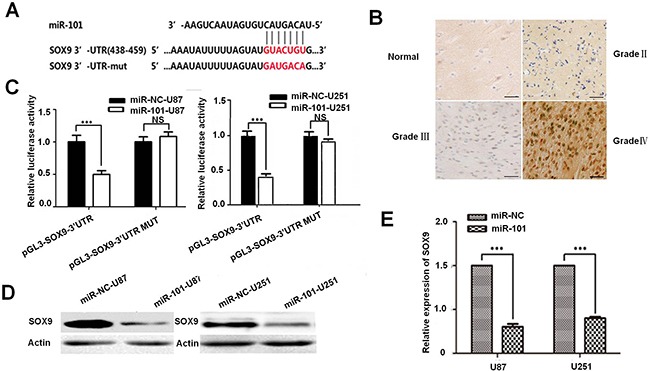
SOX9 is a direct target of miR-101 **A**. Predicted miR-101 target sequences in 3′UTR of SOX9 and mutant containing eight mutated nucleotides in 3′UTR of SOX9 (SOX9-mut). **B**. Immunohistochemistry assay detected the level of SOX9 in glioma (II-III) tissue and the normal brains tissue. **C**. U87MG and U251MG cells were co-transfected with miR-101 and luciferase reporters containing either the predicted miRNA target site in SOX9 3′UTR or its corresponding mutant form, the values obtained from the Has-miR-101 vector and PGL3 were set as 100%. **D**. Western blot analysis for the SOX9 expression in U87MG and U251MG cells. **E**. qRT-PCR analysis of SOX9 expression in U87MG and U251MG cells transfected with miR-101 or negative control.

Bioinformatics methods were adopted to predict the potential targets of miR-101 in human GBM. The TargetScan Program suggested that the 3′UTR region of the SOX9 gene containing the binding sites of miR-101 (Figure 3A), and the expression level of SOX9 in glioma (II-III) tissue was higher than that of the normal brains tissue (Figure 3B and Supplementary Figure 5). Furthermore, qRT-PCR analysis showed that SOX9 was obviously down-regulated in miR-101-U87 tumor compared with the miR-NC-U87 tumor in tumor xenograft model (Supplementary Figure 3), indicating that SOX9 might be a potential target gene of miR-101. In order to test the regulating manner between miR-101 and SOX9, we used qRT-PCR and Western blotting to compare the expression level of SOX9 in the two glioma cell lines transfected with miR-101 or miR-control as shown in Figure 3C. Both mRNA level and protein level of SOX9 was obviously decreased upon miR-101 overexpression (Figure 3E and 3D). Then we constructed a luciferase reporter plasmid containing the 3′UTR of SOX9. We found that the luciferase activity in the Luc-SOX9-UTR-transfected cells was prominent decreased compared with the luciferase activity in the miR-101 target site mutant SOX9 3′UTR and negative control cells (Figure 3C). All these results suggested that SOX9 was a direct target of miR-101 in glioma. We further used immunofluorescence to compare the SOX9 expression between miR-control U87MG and miR-101-U87MG cells (Supplementary Figure 2). The results showed that overexpression of miR-101 only reduced the SOX9 expression level but did not change the SOX9 expression pattern (Figure 3E).

### The tumor suppressing function of SOX9 *in vitro* and *in vivo*

**Figure 4:**
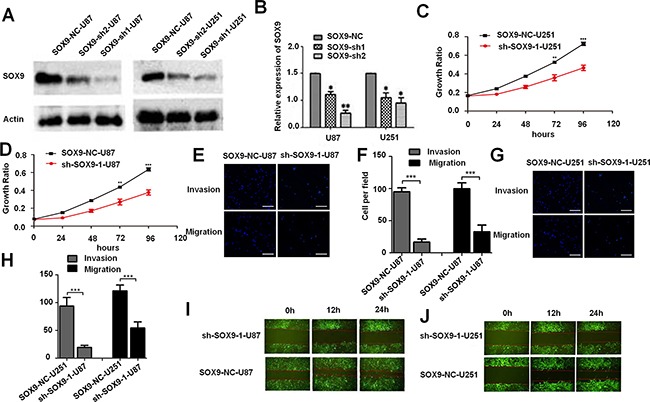
The tumor suppressing function of SOX9 *in vitro* and *in vivo* **A**. Western blotting analysis for the knockdown efficiency of SOX9 expression in U87MG and U251MG glioma cell lines. **B**. qRT-PCR analysis of SOX9 expression in U87 MG and U251MG glioma cell lines transfected with sh-SOX9-1, sh-SOX9-2, or respective negative control. **C and D**. MTT assays tested growth curves after knockdowning SOX9 in sh-SOX9-U251MG and sh-SOX9-U87MG cells. **E**. Transwell assay detected cell invasion and migration of SOX9-NC-U87MG and sh-SOX9-1-U87M cells. **F**. Quantitative analysis the invasion and migration of SOX9-NC-U87MG and sh-SOX9-1-U87M cells from three independent experiments. **G**. Trans-well assay detected cell invasion and migration in SOX9-NC-U251MG and sh-SOX9-1-U251MG cells. **H**. Quantitative analysis the invasion and migration of SOX9-NC-U251MG and sh-SOX9-1-U251MG cells from three independent experiments. **I. and J**. The wound healing assay detected cell migration in sh-SOX9-1-U251MG, NC-U251MG, sh-SOX9-1-U87MG and NC-U87MG cells, representative images were taken at different time points.

To acknowledge the targeting relationship between SOX9 and miR-101 in GBM, we also studied the SOX9 function in glioma. SOX9 was silenced in U87MG and U251MG by sh-SOX9-1 and sh-SOX9-2. The result showed that sh-hSOX9-1 showed a better knockdown efficiency (Figure 4A and 4B). MTT assay, wound healing assays and trans-well assay were used to test the effect of SOX9 on glioma cells. U87MG and U251MG glioma cell lines were infected with a lentivirus encoding the mature sequence of sh-hSOX9-1. First, MTT assay was used to examine the effect of SOX9 on U251 MG and U87MG cells proliferation. As shown in Figure 4C and 4D, the cell proliferation rate was significantly decreased in the miR-101 group compared with that of control group at 24 to 96 hours after transfection (*P < 0.05). Subsequently, trans-well assay illustrated that the invasion ability and migration ability was suppressed prominently by SOX9 silencing both in U87MG and U251MG cells (Figure 4E-4H). Wound-healing assay was used to detect the effect of SOX9 on cell migration (Figure 4I and 4J), and the SOX9-KD-U251MG and SOX9-KD-U87MG exhibited slower migration compared to the SOX9-control cells, respectively. Furthermore, SOX9 silencing also markedly inhibited mice xenograft tumor growth (Supplementary Figure 4), indicating that SOX9 was essential for glioma cell proliferation both *in vitro* and *in vivo*.

## DISCUSSION

Glioma is one of the most common primary malignant tumors in the brain parenchyma. Malignant glioma has poor prognosis despite the continuous progress in therapeutic technologies, including surgery, radiotherapy, photodynamic therapy, and chemotherapy. The high incidence and mortality of glioma prompt us to search for new therapeutic strategies. The miRNAs are kind of endogenous non - coding RNA, they can negatively regulate the target genes expression on post - transcriptional level through binding to the 3′UTR of their mRNA [33]. The deregulation of miRNAs had been found in many human cancers, such as ovarian carcinoma [34], lung cancer [35,36], liver cancer [37], colon cancer [38, 39] and GBM [40]. MiRNA deregulation has become a new characteristic of malignant tumor, so some specific miRNAs are potentially novel biomarkers of cancer diagnosis and prognosis [41–45].

MiR-101 has been reported to be down-regulated in several human cancers. Study indicated that miR-101 could repress lung cancer invasion and proliferation by inhibiting interaction of fibroblasts and cancer cells by directly targeting CXCL12 [27]. MiR-101 exercises its biological function in multiple cancer types by interating with CXCR7 [29], CDK8 [30], EZH2 level [31, 47] and CPEB1 [32]. In GBM, research showed that miR-101 could act as a tumor suppressor by targeting Kruppel-like Factor 6 in glioblastom a stem cells [46]. Furthermore, miR-101 could reverse the hypomethylation of the LMO3 promoter in glioma cells [48]. In a word, miR-101 was an important regulator in different cancers including malignant glioma.

**Figure 5:**
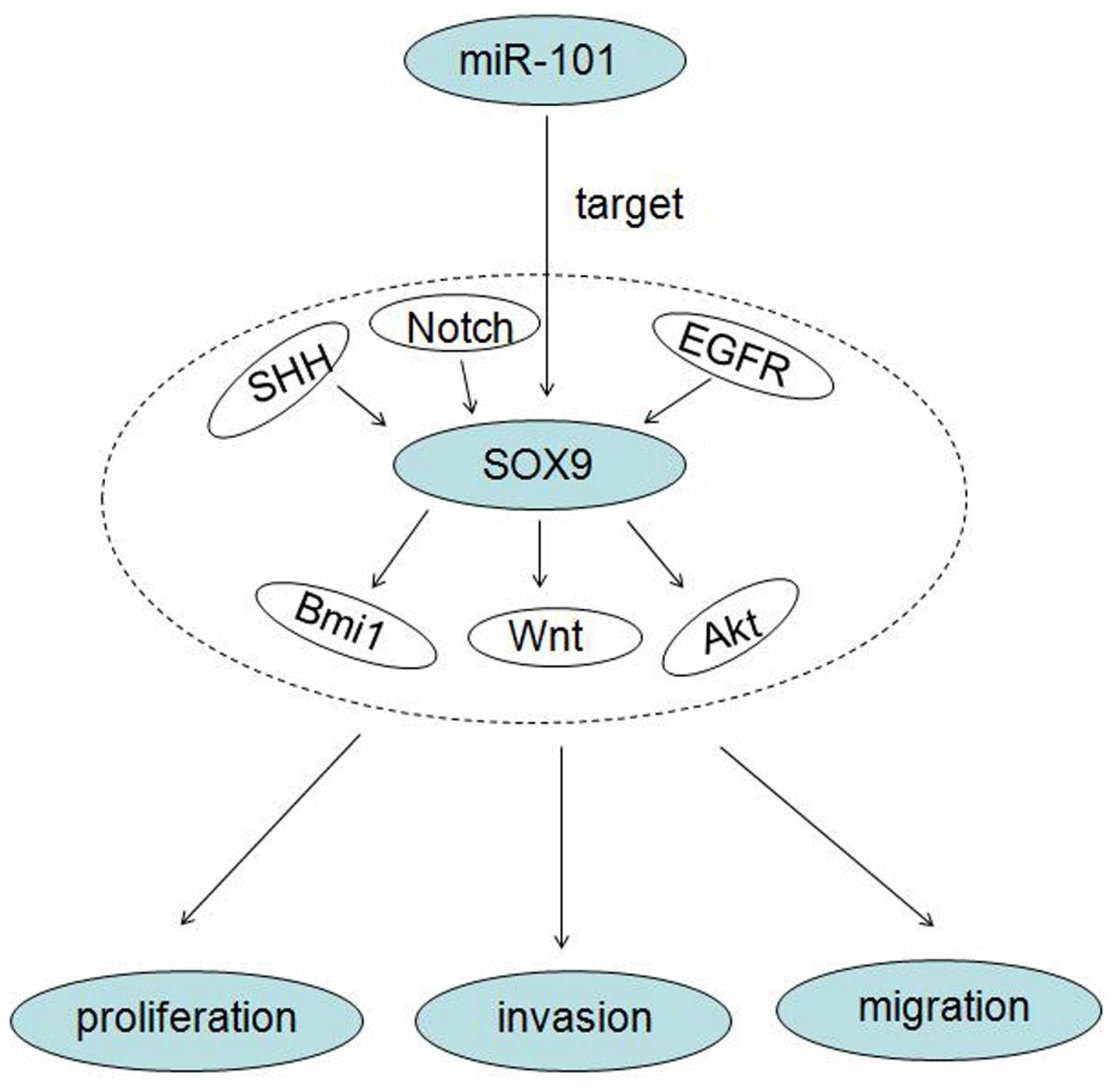
A working model of miR-101 inhibits glioma cell proliferation, invasion and migration by targeting SOX9

It is noteworthy that a study illustrated that miR-101 directly targets SOX9 in human hepatocellular carcinoma (HCC), and miR-101 could suppress SOX9-dependent tumorigenicity and promotes favorable prognosis of HCC [49]. While, SOX9 is a high mobility group box transcription factor which plays critical roles during embryogenesis, differentiation, tumor initiation, invasion and stem cell self-renewal [50, 51]. These studies prompt that SOX9 might involves in miR-101 tumor suppressing process. Therefore, we investigated the relationship between SOX9 and miR-101 in glioma in this study. Our data confirmed that miR-101 could inhibit proliferation, migration and invasion of human glioblastoma by directly targeting SOX9. Results also showed that SOX9 was essential for glioma tumorigenesis both *in vitro* and *in vivo*. SOX9 is reported to be regulated by EGFR [52], Notch [53], SHH [54], and in turn regulate Akt [55], Wnt [56], BMI1 [57] pathways. So we conclude that miR-101 and SOX9 regulation axis regulates proliferation, migration and invasion of human GBM by regulating Akt, Wnt, BMI1 signal pathway (Figure 5). This study suggests us that miR-101 and SOX9 are key regulators in human glioblastoma and provides new therapeutic targets for glioma therapy.

## MATERIALS AND METHODS

### Cell cultures and human tissue Samples

Human glioma cell lines U251MG and U87MG were purchased from the Chinese Academy of Sciences Cell Bank in 2012, A172, T98 and HEB Normal glial cell lines were purchased from Beijing ChuangLian biotechnology company (Beijing, China). The authenticity of cancer cell lines was tested by short tandem repeat profiling. All cancer cells were maintained in high glucose DMEM (Invitrogen, Grand Island, NY, USA) supplemented with 10% FBS (GIBCO, USA), 100 units/mL penicillin (NCPC, Shijiazhuang, China) and 100 μg/mL streptomycin (NCPC, Shijiazhuang, China), and incubated in 5% CO_2_ at 37°C. Specimens of human glioma tissues were collected from 25 patients treated in Tangdu Hospital of Fourth Military Medical University, P. R. China. The normal brain tissue specimens were taken from 5 patients who have encountered with traumatic brain injuries. The study was approved by the Research Ethics Committee of Tangdu Hospital of Fourth Military Medical University, P. R. China. All patients involved in this study have signed the informed consent be beforehead, and all specimens were handled anonymous processing according to ethical and legal standards.

### Isolation of total RNA and real-time PCR analysis

The expression level of miR-101 in glioma cells, glioma tissues and traumatic brain injuries tissues was measured by real-time quantitative RT-PCR (qRT-PCR). Total RNA was isolated from frozen samples and cells using Trizol reagent (Invitrogen, CA, USA) under the guidance of manufacturer's protocol. RNA was treated with RNase-free DNase I (Roche, Switzerland). Then, BcaBest RNA PCR kit (TaKaRa, Dalian, China) was used to synthesize the cDNA according to the manufacturer's protocol. All primers were synthesized by Shanghai Sangon Technology co., LTD. Quantitative RT-PCR was carried out by the iQ5 Multicolor Real-Time PCR Detection System (Bio-Rad) with Real-time PCR Master Mix (SYBR Green).

### Vector construction and transfection

To construct the miR-101 overexpression vector, the primers of miR-101 were synthesized by Shanghai Generay Biotech Co., Ltd. Primer sets (5′-3′) used for amplifying the miR-101 were as follows, (Forward primer: CCTGAATTCATTCTAATTTAATTCAACTGG; Reverse primer: TATGGATCCTCAGCACAACATGGCTGCAC), containing *EcoR I* and *BamH I* respectively. Amplified miR-101 was then subcloned into pCDH1vector between *EcoRI* and *BamH I* sites (Promega, Madison, WI, USA). Glioma cells were infected with a lentivirus encoding the miR-101 mimic oligonucleotide (200nM), negative control (NC) and fluorescent GAPDH positive control (GenePharma, Shanghai, China). 10 days after infection, the cells were harvested and RNA was extracted for qRT-PCR analysis after filtered with 5ug/ml purine toxins. We also constructed SOX9 knock down stable cell lines. The premade lentiviral SOX9 short hairpin RNA (shRNA) constructs and a shNC constructs were purchased from GenePharma (Shanghai, China). The sequences are sh-SOX9-1: GCATCCTTCAATTTCTGTATA, sh-SOX9-2: CTCCACCTTCACCTACATGAA. The interference fragment was subcloned into LV3 vector, lentivirus product were also purchased from GenePharma (Shanghai, China). The DNA sequence of SOX9 3′-UTR including the miR-101 binding site was amplified by PCR using wild type SOX9 primers GAATTCTCAGTGGCCAGGCCAACCTTV-5′ and CATATGAAACTGATCACATAACACAA-3′, and subsequently cloned into the PGL3-luc vector (Promega, USA). The predicted miR-101 target site GUACUGU was mutated into GAUGACA by site-directed mutagenesis. The SOX9 Wild type primers used were GAATT CTCAGTGGCCAGGCCAACCTTV-5′ and CATATG AAACTGATCACATAACACAA-3′, mutagenic primers used were ATATTTTTAGTATGATGACAGTATGATTCAT-5′ and ATGAATCATACTGTCATCATACTAAAAATAT-3′.

### MTT assay

The MTT assays were performed as described before [23]. In brief, 1 × 10^4^ cells/well was seeded in 96-well plates with 200 μL culture medium. After treatments, the medium was replaced with 200 μL DMEM/FBS containing 0.5 mg/mL MTT and incubated at 37°C for 4 h. The supernatant was then discarded, and the cells were lysed in 200 μL DMSO for 10 min at 37°C. The optical density (OD) values were measured at 490 nm (SpectraMax 190; Molecular Devices Sunnyvale, CA, USA).

### Cell invasion and migration assays

Cells (2.5 × 10^5^) were suspended in 250 μL serum-free DMEM and seeded in the top chambers of 24-well transwell plates (Corning Inc., Corning, NY, USA) coated with 30 μL Matrigel (BD Biosciences, Franklin Lakes, NJ, USA). The bottom chambers of the trans-well plates were filled with 600 μL DMEM containing 10% FBS. Cells were allowed to migrate for 48 hours (invasion assay) at 37°C [24]. After migration, cells in the top chambers were removed using a cotton swab, and the cells which migrated to the bottom chambers were fixed in 4% paraformaldehyde (PFA; Sigma-Aldrich) and stained with Hoechst staining. The fixed and stained cells were counted in five independent fields under a light microscope. At least three chambers were counted for each experiment. For the migration assay, a similar protocol was followed except for the replacement of the top chamber of the tran-swell plate with an uncoated chamber. The culture medium in the bottom chamber was replaced with DMEM containing 2.5% FBS, and cells were allowed to migrate for 8 hours.

### Wound healing assays

Cells were seeded in 6-well plates and cultured until they reached confluence, then a wound was created by manually scraping the cell monolayer with a sterile 200 μL pipette tip. Cells were washed twice with PBS to remove the floating cells, and then incubated in DMEM supplemented with 1% FBS. Cell migration was observed at three preselected time points (0, 12, and 24 hours). Images were acquired with a Nikon DS-5M Camera System mounted on a phase-contrast Leitz microscope.

### Immunohistochemistry assay

For immunohistochemistry (IHC), 8 μm sections of formalin-fixed and paraffin embedded brain tissues were first de-waxed and rehydrated before antigen retrieval. The SOX9-antibody (1:100 dilution; Abcam, Cambridge, USA) and Ki67-antibody (1:100; Roche, Basel, Switzerland) were used for this study. After incubation with the primary antibodies, the cells were rinsed and incubated for one hour with Biotin-labeled secondary antibodies at room temperature (Molecular Probes 1:800). Nuclei were stained by Hematoxylin. Stained sections were examined under a light microscope and the positive cells in five high power fields (1×200) were counted for statistic study.

### Western blotting

The total cell lysates were dissolved in high KCl lysis buffer (10 mM Tris-HCl, pH 8.0, 140 mM NaCl, 300 mM KCl, 0.5% Triton X-100,1 mM EDTA, and 0.5% sodium deoxycholate) with complete protease inhibitor cocktail (Roche). The protein concentrations were determined by a protein assay kit (Bio-Rad, Hercules, CA). The Western blot assay has been previously described [25]. SOX9-antibody was used as primary antibodies. Immunoreactivity was visualized by enhanced chemiluminescence amplification according to the manufacturer's protocol (GE healthcare, Buckinghamshire, UK).

### Luciferase assay

The Hsa-miR-101 vector (GenePharma Co.) and PGL3, PGL3-SOX9 3′-UTR, PGL3-SOX9 3′-UTR-mut were cotransfected into HEK293T cells. Twenty four hours after transfection, cell lysates were harvested. The luciferase activity was measured using the Dual-Luciferase Reporter Assay System (Promega, USA).

### Animal experiments

All animal experiments were approved by the Research Ethics Committee of Tangdu Hospital of Fourth Military Medical University, P. R. China. Athymic/nude immunocompromised mice were purchased from Fourth Military Medical University (Shanxi, China) and breeding colonies were maintained in our animal facility under standard conditions. Xenografted transplantation of glioma cells into athymic/nude immunocompromised mice was performed as previously described [26]. There are miR-NC group and miR-101 group for the U87MG cell lines. After pre-transplant preparation of the recipient mice and anesthesia with 10% chloral hydrate, isolated miR-101-U87MG cells (3.0×10^6^ in 5 mL PBS) and their respective control cells were implanted into the left and right flanks (cells per flank) of nude mice by subcutaneous injection to establish the xenograft model. The weight change of each animal was measured daily. Tumor volumes were determined by measuring the length (a) and the width (b). The tumor volume (V) was calculated according to the formula V = ab^2^/2 [27].

### Statistical analysis

Independent samples were analyzed by using one-sided unpaired Student's t tests with SPSS17.0 for windows (SPSS Inc., Chicago, IL, USA). All statistical results from the quantitative analysis of the *in vitro* experiments are presented as means ± SEM, as specified in the figure legends. *p* values < 0.05 were considered statistically significant.

## SUPPLEMENTARY FIGURES



## References

[1] Buckner JC, Brown PD, O’Neill BP, Meyer FB, Wetmore CJ, Uhm JH (2007). Central nervous system tumors. Mayo Clin Proc.

[2] Louis DN, Ohgaki H, Wiestler OD, Cavenee WK, Burger PC, Jouvet A, Scheithauer BW, Kleihues P (2007). The 2007 WHO classification of tumours of the central nervous system. Aeta Neuro Pathol.

[3] Mason WP, Caimcross JG (2005). Drug Insight: temozolomide as a treatment for malignant glioma - impact of a recent trial. Nat Clin Pract Neurol.

[4] Stupp R, Mason WP, van den Bent MJ, Weller M, Fisher B, Taphoorn MJ, Belanger K, Brandes AA, Marosi C, Bogdahn U, Curschmann J, Janzer RC, Ludwin SK (2005). European Organisation for Research and Treatment of Cancer Brain Tumor and Radiotherapy Groups; National Cancer Institute of Canada Clinical Trials Group. Radiotherapy plus concomitant and adjuvant temozolomide for glioblastoma. N Engl J Med.

[5] Kleber S, Sancho-Martinez I, Wiestler B, Beisel A, Gieffers C, Hill O, Thiemann M, Mueller W, Sykora J, Kuhn A, Schreglmann N, Letellier E, Zuliani C (2008). Yes and PI3K bind CD95 to signal invasion of glioblastoma. Cancer Cell.

[6] Ohgaki H, Kleihues P (2005). Population-based studies on incidence, survival rates, and genetic alterations in astrocytic and oligodendroglial gliomas. J Neuropathol Exp Neurol.

[7] Ohgaki H, Dessen P, Jourde B, Horstmann S, Nishikawa T, Di Patre PL, Burkhard C, Schüler D, Probst-Hensch NM, Maiorka PC, Baeza N, Pisani P, Yonekawa Y (2005). Genetic pathways to glioblastoma: a population-based study. Cancer Res.

[8] Stupp R, Mason WP, van den Bent MJ, Weller M, Fisher B, Taphoorn MJ, Belanger K, Brandes AA, Marosi C, Bogdahn U, Curschmann J, Janzer RC, Ludwin SK (2005). Radiotherapy plus concomitant and adjuvant temozolomide for glioblastoma. N Engl J Med.

[9] Singh SK, Clarke ID, Terasaki M, Bonn VE, Hawkins C, Squire J, Dirks PB (2003). Identification of a cancer stem cell in human brain tumors. Cancer Res.

[10] Singh SK, Hawkins C, Clarke ID, Squire JA, Bayani J, Hide T, Henkelman RM, Cusimano MD, Dirks PB (2004). Identification of human brain tumour initiating cells. Nature.

[11] Bao S, Wu Q, McLendon RE, Hao Y, Shi Q, Hjelmeland AB, Dewhirst MW, Bigner DD, Rich JN (2006). Glioma stem cells promote radioresistance by preferential activation of the DNA damage response. Nature.

[12] Hong B, Wiese B, Bremer M, Heissler HE, Heidenreich F, Krauss JK, Nakamura M (2013). Multiple microsurgical resections for repeated recurrence of glioblastoma multiforme. Am J Clin Oncol.

[13] Becker KP, Yu J (2012). Status quo-standard-of-care medical and radiation therapy for glioblastoma. Cancer J.

[14] Yaman E, Coskun U, Ozturk B, Buyukberber S, Kaya AO, Coskun O, Buyukberber N, Yildiz R, Benekli M (2009). Opportunistic cytomegalovirus infection in a patientreceiving temozolomide for treatment of malignant glioma. J Clin Neurosci.

[15] Corsten MF, Miranda R, Kasmieh R, Krichevsky AM, Weissleder R, Shah K (2007). MicroRNA-21 knockdown disrupts glioma growth in vivo and displays synergistic cytotoxicity with neural precursor cell delivered S-TRAIL in human gliomas. Cancer Res.

[16] Gabriely G, Wurdinger T, Kesari S, Esau CC, Burchard J, Linsley PS, Krichevsky AM (2008). MicroRNA 21 promotes glioma invasion by targeting matrix metalloproteinase regulators. Mol Cell Biol.

[17] Bartel DP (2004). MicroRNAs: genomics, biogenesis, mechanism, andfunction. Cell.

[18] Burnet NG, Lynch AG, Jefferies SJ, Price SJ, Jones PH, Antoun NM, Xuereb JH, Pohl U (2007). High grade glioma: imaging combined with pathological grade defines management and predicts prognosis. Radiother Oncol.

[19] Liu X, Fortin K, Mourelatos Z (2008). MicroRNAs: biogenesis and molecular functions. Brain Pathol.

[20] Tavallaie R, De Almeida SR, Gooding JJ (2014). Toward biosensors for the detection of circulating microRNA as a cancer biomarker: an overview of the challenges and successes. Wiley Interdiscip Rev Nanomed Nanobiotechnol.

[21] Ohgaki H, Dessen P, Jourde B, Horstmann S, Nishikawa T, Di Patre PL, Burkhard C, Schüler D, Probst-Hensch NM, Maiorka PC, Baeza N, Pisani P, Yonekawa Y (2005). Genetic pathways to glioblastoma: a population-based study. Cancer Res.

[22] Furnari FB, Fenton T, Bachoo RM, Mukasa A, Stommel JM, Stegh A, Hahn WC, Ligon KL, Louis DN, Brennan C, Chin L, DePinho RA, Cavenee WK (2007). Malignant astrocytic glioma: genetics, biology, and paths to treatment. Genes Dev.

[23] Han L, Zhang A, Wang H, Pu P, Jiang X, Kang C, Chang J (2010). Tat-BMPs-PAMAM conjugates enhance therapeutic effect of small interference RNA on U251 glioma cells in vitro and in vivo. Hum Gene Ther.

[24] Tu Y, Gao X, Li G, Fu H, Cui D, Liu H, Jin W, Zhang Y (2013). MicroRNA-218 inhibits glioma invasion, migration, proliferation, and cancer stem-like cell self-renewal by targeting the polycomb group gene Bmi1. Cancer Res.

[25] Mi YJ, Hou B, Liao QM, Ma Y, Luo Q, Dai YK, Ju G, Jin W (2012). Amino-Nogo-A antagonizes reactive oxygen species generation and protects immature primary cortical neurons from oxidative toxicity. Cell Death Differ.

[26] Cheng L, Wu Q, Guryanova OA, Huang Z, Huang Q, Rich JN, Bao S (2011). Elevated invasive potential of glioblastoma stem cells. Biochem Biophys Res Commun.

[27] Xia H, Yan Y, Hu M, Wang Y, Dai Y, Chen J, X Di G Chen, Jiang X (2012). MiR-218 sensitizes glioma cells to apoptosis and inhibits tumorigenicity by regulating ECOP-mediated suppression of NF-kappaB activity. Neuro-oncology.

[28] Zhang J, Liu J, Liu Y, Wu W, Li X, Wu Y, Chen H, Zhang K, Gu L (2015). miR-101 represses lung cancer by inhibiting interaction of fibroblasts and cancer cells by down-regulating CXCL12. Biomed Pharmacother.

[29] Li JT, Jia LT, Liu NN, Zhu XS, Liu QQ, Wang XL, Yu F, Liu YL, Yang AG, Gao CF (2015). MiRNA-101 inhibits breast cancer growth and metastasis by targeting CX chemokine receptor 7. Oncotarget.

[30] Li M, Tian L, Ren H, Chen X, Wang Y, Ge J, Wu S, Sun Y, Liu M, Xiao H (2015). MicroRNA-101 is a potential prognostic indicator of laryngeal squamous cell carcinoma and modulates CDK8. J Transl Med.

[31] Vella S, Pomella S, Leoncini PP, Colletti M, Conti B, Marquez VE, Strillacci A, Roma J, Gallego S, Milano GM, Capogrossi MC, Bertaina A, Ciarapica R, Rota R (2015). MicroRNA-101 is repressed by EZH2 and its restoration inhibits tumorigenic features in embryonal rhabdomyosarcoma. Clin Epigenetics.

[32] Xiaoping L, Zhibin Y, Wenjuan L, Zeyou W, Gang X, Zhaohui L, Ying Z, Minghua W, Guiyuan L (2013). CPEB1, a histone-modified hypomethylated gene, is regulated by miR-101 and involved in cell senescence in glioma. Cell Death Dis.

[33] Ambros V (2001). microRNAs: tiny regulators with great potential. Cell.

[34] Nam EJ, Yoon H, Kim SW, Kim H, Kim YT, Kim JH, Kim JW, Kim S (2008). MicroRNA expression profiles in serous ovarian carcinoma. Clin Cancer Res.

[35] Zhu D, Chen H, Yang X, Chen W, Wang L, Xu J, Yu L (2014). Decreased microRNA-224 and its clinical significance in non-small cell lung cancer patients. Diagn Patho.

[36] Yang Y, Meng H, Peng Q, Yang X, Gan R, Zhao L, Chen Z, Lu J, Meng QH (2014). Downregulation of microRNA-21 expression restrains non-small cell lung cancer cell proliferation and migration through upregulation of programmed cell death. Cancer Gene Ther.

[37] Yang N, Ekanem NR, Sakyi CA, Ray SD (2015). Hepatocellular carcinoma and microRNA: New perspectives on therapeutics and diagnostics. Adv Drug Deliv Rev.

[38] Chen P, Wang BL, Pan BS, Guo W (2014). MiR-1297 Regulates the Growth, Migration and Invasion of Colorectal Cancer Cells by Targeting Cyclo-oxygenase-2. Asian Pac J Cancer Prev.

[39] Zhou MK, Liu XJ, Zhao ZG, Cheng YM (2015). MicroRNA-100 functions as a tumor suppressor by inhibiting Lgr5 expression in colon cancer cells. Mol Med Rep.

[40] Turner JD, Williamson R, Almefty KK, Nakaji P, Porter R, Tse V, Kalani MY (2010). The many roles of microRNAs in brain tumor biology. Neurosurg Focus.

[41] Yu SL, Chen HY, Chang GC, Chen CY, Chen HW, Singh S, Cheng CL, Yu CJ, Lee YC, Chen HS, Su TJ, Chiang CC, Li HN (2008). MicroRNA signature predicts survival and relapse in lung cancer. Cancer Cell.

[42] Shenouda SK, Alahari SK (2009). MicroRNA function in cancer: oncogene or a tumor suppressor. Cancer Metastasis Rev.

[43] Cho WC (2010). MicroRNAs: potential biomarkers for cancer diagnosis, prognosis and targets for therapy. Int J Biochem Cell Biol.

[44] Liu N, Tu Y (2015). Systematic Review of MicroRNAs and its Therapeutic Potential in Glioma. Cancer Transl Med.

[45] Zhang P, Pang X, Tu Y (2015). Thioredoxin-interacting Protein as a Common Regulation Target for Multiple Drugs in Clinical Therapy/Application. Cancer Transl Med.

[46] Yao YL, Ma J, Wang P, Xue YX, Li Z, Zhao LN, Li ZQ, Feng TD, Liu YH (2015). miR-101 Acts as a Tumor Suppre ssor by Targeting Krup pel-like Facto r 6 in Gli oblastom a Stem Cells. CNS Neurosci Ther.

[47] Smits M, Nilsson J, Mir SE, van der Stoop PM, Hulleman E, Niers JM, de Witt Hamer PC, Marquez VE, Cloos J, Krichevsky AM, Noske DP, Tannous BA, Würdinger T (2010). miR-101 is down-regulated in glioblastoma resulting in EZH2- induced proliferation, migration, and angiogenesis. Oncotarget.

[48] Liu Lei Q, Yu Z, Xu G, Tang H, Wang W, Wang Z, Li G, Wu M (2015). MiR-101 reverses the hypomethylation of the LMO3 promoter in glioma cells. Oncotarget.

[49] Zhang Y, Guo X, Xiong L, Kong X, Xu Y, Liu C, Zou L, Li Z, Zhao J, Lin N (2012). MicroRNA-101 suppresses SOX9-dependent tumorigenicity and promotes favorable prognosis of human hepatocellular carcinoma. FEBS Lett.

[50] Sekido R, Lovell-Badge R (2009). Sex determination and SRY: down to a wink and a nudge?. Trends Genet.

[51] Larsimont JC, Youssef KK, Sánchez-Danés A, Sukumaran V, Defrance M, Delatte B, Liagre M, Baatsen P, Marine JC, Lippens S, Guerin C, Del Marmol V, Vanderwinden JM (2015). Sox9 Controls Self-Renewal of Oncogene Targeted Cells and Links Tumor Initiation and Invasion. Cell Stem Cell.

[52] Golding SE, Morgan RN, Adams BR, Hawkins AJ, Povirk LF, Valerie K (2009). Pro-survival AKT and ERK signaling from EGFR and mutant EGFRvIII enhances DNA double-strand break repair in human glioma cells. Cancer Biol Ther.

[53] Wang J, Wakeman TP, Lathia JD, Hjelmeland AB, Wang XF, White RR, Rich JN, Sullenger BA (2010). Notch promotes radioresistance of glioma stem cells. Stem Cells.

[54] Scott CE, Wynn SL, Sesay A, Cruz C, Cheung M, Gomez Gaviro MV, Booth S, Gao B, Cheah KS, Lovell-Badge R, Briscoe J (2010). SOX9 induces and maintains neural stem cells. Nat Neurosci.

[55] Ikegami D, Akiyama H, Suzuki A, Nakamura T, Nakano T, Yoshikawa H, Tsumaki N (2011). Sox9 sustains chondrocyte survival and hypertrophy in part through Pik3ca-Akt pathways. Development.

[56] Bastide P, Darido C, Pannequin J, Kist R, Robine S, Marty-Double C, Bibeau F, Scherer G, Joubert D, Hollande F, Blache P, Jay P (2007). Sox9 regulates cell proliferation and is required for Paneth cell differentiation in the intestinal epithelium. J Cell Biol.

[57] Matheu A, Collado M, Wise C, Manterola L, Cekaite L, Tye AJ, Canamero M, Bujanda L, Schedl A, Cheah KS, Skotheim RI, Lothe RA, López de Munain A (2012). Oncogenicity of the developmental transcription factor Sox9. Cancer Res.

