# Supplementary material for: MicroRNA-101 inhibits proliferation, migration and invasion of human glioblastoma by targeting SOX9
Source: Oncotarget. 2016 Nov 30;8(12):19244–54. doi: 10.18632/oncotarget.13706 (PMC5386681; doi:10.18632/oncotarget.13706)
Supplement: Supplementary file 1 [file oncotarget-08-19244-s001.pdf]

MicroRNA-101 inhibits proliferation, migration and invasion of human glioblastoma by targeting SOX9

SUPPLEMENTARY FIGURES

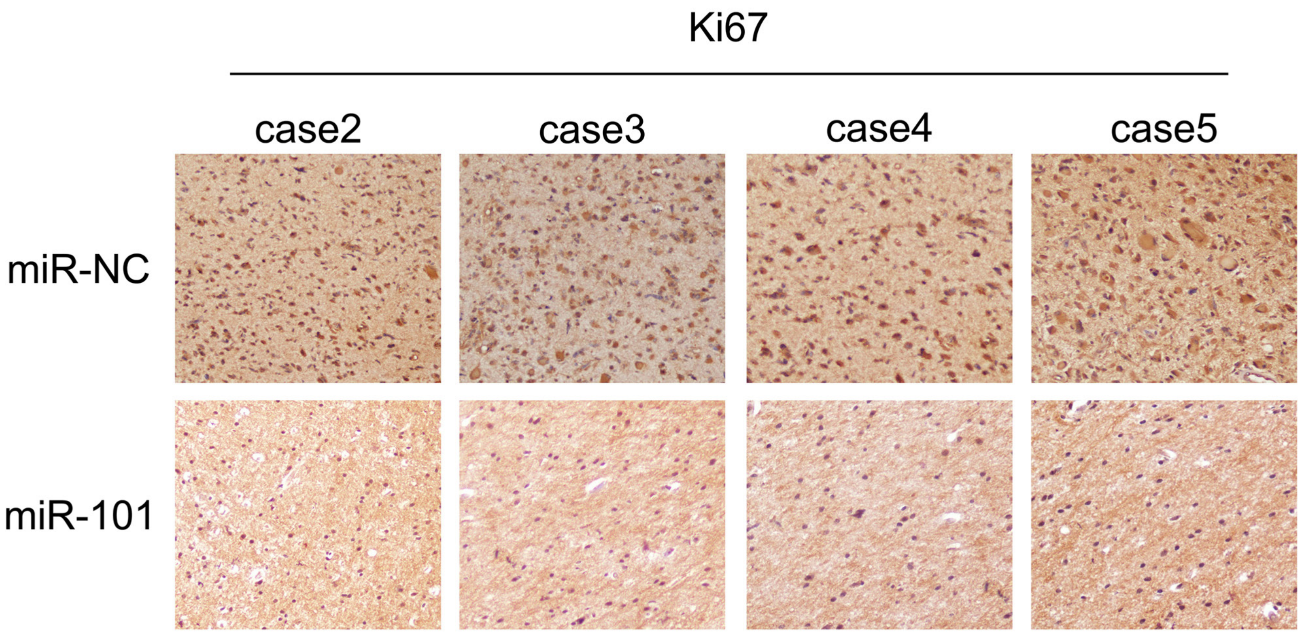

Supplementary Figure 1: Ki-67 staining of the rest 4 tumor xenograft model cases.

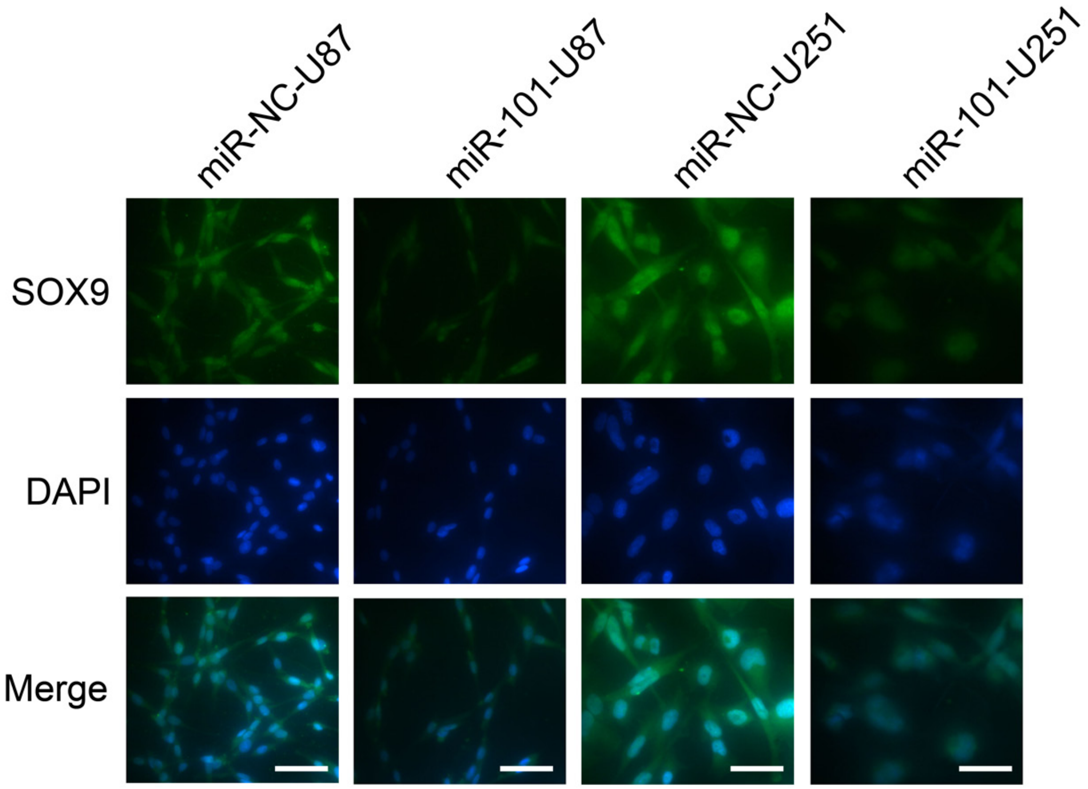

Supplementary Figure 2: Expression pattern of SOX9 in miR-NC and miR-101 transfected U87MG and U251MG cells.

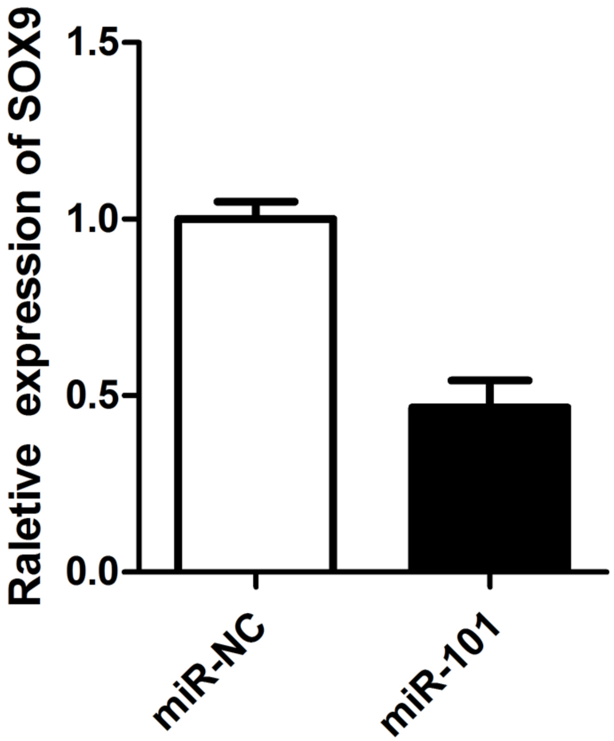

Supplementary Figure 3: Expression level of SOX9 in miR-101-U87 tumor xenograft model sample by qRT-PCR.

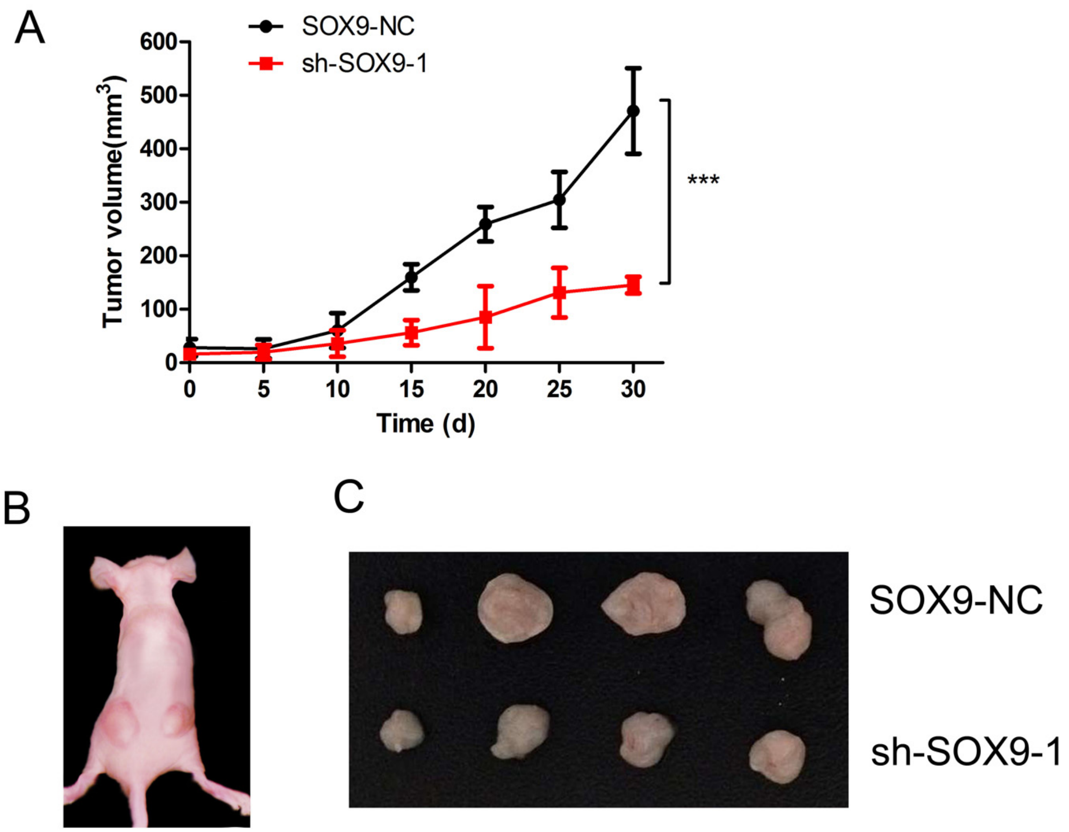

Supplementary Figure 4: Tumor xenograft model to detect effect of SOX9 silencing in glioma tumor growth. **A.** Determination of the tumor growth, tumor volume was calculated every five days after injection (n = 5). $^{***}p < 0.001$. **B.** Representative image for tumor growth is shown. Nude mice were subcutaneously injected with 3.0×10^6 cells per flank SOX9-NC and shSOX9-1 stable transfected U87MG cells. **C.** Representative images of the tumor at 30 days.

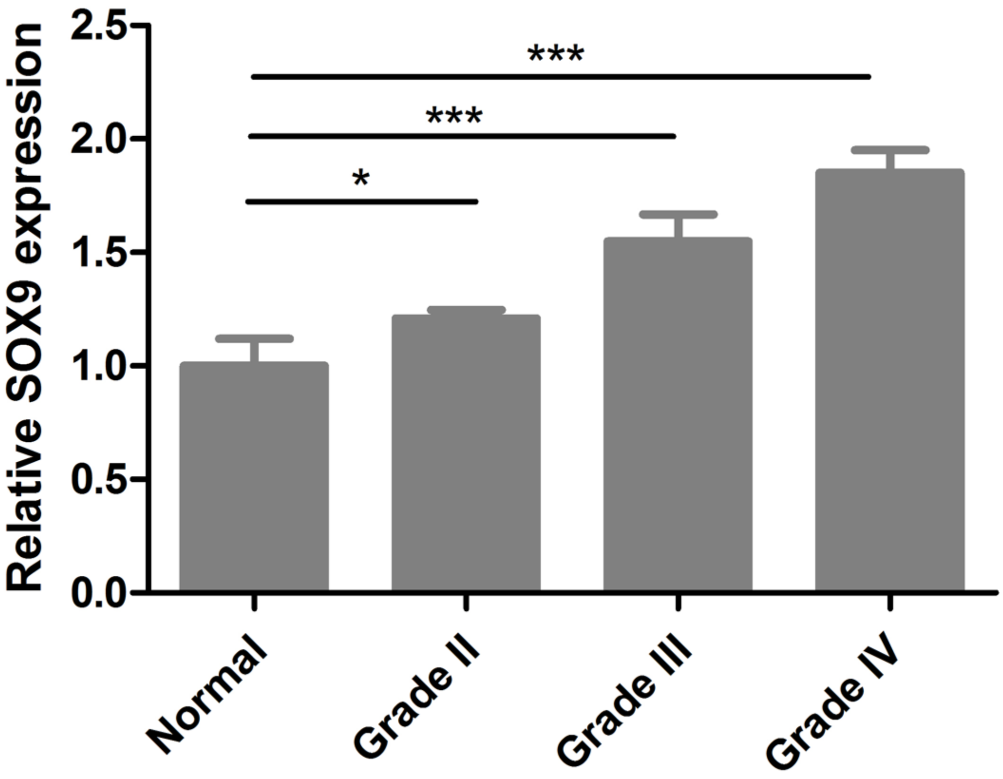

Supplementary Figure 5: SOX9 expression levels were significantly increased according to samples patients' grades.
